# Sorsby Fundus Dystrophy Mutation in Tissue Inhibitor of Metalloproteinase 3 (TIMP3) promotes Choroidal Neovascularization via a Fibroblast Growth Factor-dependent Mechanism

**DOI:** 10.1038/s41598-019-53433-6

**Published:** 2019-11-22

**Authors:** Jian Hua Qi, Brent Bell, Rupesh Singh, Julia Batoki, Alyson Wolk, Alecia Cutler, Nicholas Prayson, Mariya Ali, Heidi Stoehr, Bela Anand-Apte

**Affiliations:** 1 Department of Ophthalmology, Cleveland Clinic Lerner College of Medicine, Cole Eye Institute, Cleveland Clinic Foundation, Cleveland, OH USA; 2 Institute of Human Genetics, University of Regensburg, Regensburg, Germany; 3 Department of Molecular Medicine, Cleveland Clinic Lerner College of Medicine, Cleveland Clinic Foundation, Cleveland, OH USA

**Keywords:** Extracellular signalling molecules, Experimental models of disease

## Abstract

Choroidal neovascularization (CNV) leads to loss of vision in patients with Sorsby Fundus Dystrophy (SFD), an inherited, macular degenerative disorder, caused by mutations in the Tissue Inhibitor of Metalloproteinase-3 (*TIMP3*) gene. SFD closely resembles age-related macular degeneration (AMD), which is the leading cause of blindness in the elderly population of the Western hemisphere. Variants in *TIMP3* gene have recently been identified in patients with AMD. A majority of patients with AMD also lose vision as a consequence of choroidal neovascularization (CNV). Thus, understanding the molecular mechanisms that contribute to CNV as a consequence of TIMP-3 mutations will provide insight into the pathophysiology in SFD and likely the neovascular component of the more commonly seen AMD. While the role of VEGF in CNV has been studied extensively, it is becoming increasingly clear that other factors likely play a significant role. The objective of this study was to test the hypothesis that basic Fibroblast Growth Factor (bFGF) regulates SFD-related CNV. In this study we demonstrate that mice expressing mutant TIMP3 (*Timp3*^*S179C/S179C*^) showed reduced MMP inhibitory activity with an increase in MMP2 activity and bFGF levels, as well as accentuated CNV leakage when subjected to laser injury. S179C mutant-TIMP3 in retinal pigment epithelial (RPE) cells showed increased secretion of bFGF and conditioned medium from these cells induced increased angiogenesis in endothelial cells. These studies suggest that S179C-TIMP3 may promote angiogenesis and CNV via a FGFR-1-dependent pathway by increasing bFGF release and activity.

## Introduction

The sprouting of new blood vessels from pre-existing vasculature, termed angiogenesis is crucial for a number of physiological processes^1^. It occurs as a cascade of events initiated by angiogenic factors.which induce the secretion of capillary basement membrane-degrading proteases followed by migration, proliferation and tube formation of endothelial cells (ECs), and is intricately regulated by a temporal and spatial balance of inducers and inhibitors^2–4^. Uncontrolled, neovascularization contributes to a variety of malignant, ischemic, infectious and immune disorders^2,3,5^. Vascular endothelial growth factor (VEGF-A) and basic fibroblast growth factor (bFGF or FGF-2) represent two key mediators of angiogenesis and play a critical role in both physiological and pathological angiogenesis^6–11^. VEGF-A and bFGF are potent angiogenesis inducers that mainly exhibit angiogenic effects by binding to their cognate receptor VEGF receptor-2 (VEGFR-2/KDR/Flk-1) or FGFR-1, respectively, on the surface of ECs^12,13^. Following binding of VEGF or bFGF, VEGFR-2 and FGFR-1 are dimerized and autophosphorylated, resulting in activation of signal transduction pathways and eventually in cellular responses^12–15^. In addition to interacting with VEGFR-2 and FGFR-1, VEGF-A and bFGF bind to cell membrane-associated and/or extracellular matrix (ECM)-associated heparan sulfate proteoglycans (HSPG)^16–18^. HSPGs and heparin are potent modulators of FGF activity and can protect FGF from thermal denaturation and proteolytic degradation. Binding of FGF to HSPGs provides a reservoir from which FGF can be rapidly released in response to specific triggering events^17,19,20^. Others have suggested that interaction of FGFs with HSPGs increases their receptor binding affinity by stabilizing growth factor-receptor complexes^21,22^. HSPSs and heparin may also facilitate FGFR dimerization and subsequent activation^15^.

Matrix metalloproteinases (MMPs), a family of soluble and membrane-anchored proteolytic enzymes that can degrade components of ECM, are also important regulators of angiogenesis^23–25^. Apart from clearing a path for migrating ECs by breaking down matrix components, MMPs can switch on angiogenesis by liberating matrix-bound angiogenic activators including bFGF or by increasing the bioavailability of VEGF^25,26^. Tissue inhibitor of metalloproteinase-3 (TIMP3), one of four members of a protein family that were originally classified based on their ability to inhibit MMPs^27,28^, is a naturally occurring inhibitor of angiogenesis that limits vessel density in vascular bed of tumors and curtails tumor growth^29,30^. Like other TIMPs, TIMP3 can be separated into an N-terminal domain that is responsible for MMP inhibition, and a C-terminal domain that confers specific functions such as binding to pro-MMPs, each containing six cysteine residues that form three disulfide bonds^28^. Unlike other soluble members of the TIMP family, TIMP3 is tightly sequestered in the extracellular matrix (ECM). TIMP3 is synthesized by retinal pigment epithelial (RPE) cells and deposited in Bruch’s membrane (BM) where it is a component of the ECM^27,28^. It is also present at low levels around blood vessels and localized to the choriocapillary bed matrix of human eyes^31^. Moreover, TIMP-3 is the only TIMP that can inhibit tumor necrosis factor alpha (TNF-α) converting enzyme (TACE/ADAM17), aggrecanase 1 and 2 (ADAMTS4 and ADAMTS5) and syndecan sheddase^32,33^. TIMP3 has various additional functions such as the inhibition of f VEGF binding to VEGFR-2 and the induction of apoptosis^34–36^.

Point mutations in the C-terminal domain of TIMP3, that insert single cysteines into non-conserved sites (Tyr151Cys, Glu162Lys, Tyr174Cys, Tyr177Cys, Ser179Cys, Tyr182Cys, Gly189Cys, Gly190Cys, Tyr191Cys, Ser193Cys, Tyr195Cys, Tyr198Cys and Ser204Cys), are linked to Sorsby fundus Dystrophy (SFD), an inherited form of blindness^37,38^. Although SFD is a relatively rare disease, it is of considerable interest because of its striking similarity to age-related macular degeneration (AMD), a leading cause of irreversible visual impairment and blindness in the elderly population^39–43^. New blood vessel formation from the choroid, known as choroidal neovascularization (CNV), is the hallmark of ‘wet’ AMD and is the chief cause of irreversible loss of vision due to the induction of hemorrhage, serous exudates, and/or retinal detachment^39^. While the exact molecular mechanism(s) underlying CNV is not completely understood, anti VEGF drugs have been shown to provide benefit in some patients. We have previously shown VEGF to likely play a partial role in the pathogenesis of CNV^44^. However, it is becoming increasingly evident that other yet unidentified factors are also involved. The role of bFGF in the regulation of CNV in AMD is still unclear^45^. Since SFD is one of the few examples of a single gene mutation that results in an increased neovascularization phenotype in humans, the study of the consequences of this type of mutation on TIMP3 functions would allow a molecular dissection of relevant mechanisms leading to CNV and has been the subject of recent attention^44,46–51^. In the present study, we provide evidence that bFGF may play a role in the pathogenesis of CNV in SFD via MMP2-dependent mechanisms.

## Results

### Increased vascular leakage in laser-induced CNV in *Timp3*^*S179C/S179C*^ mice

**Figure 1:**
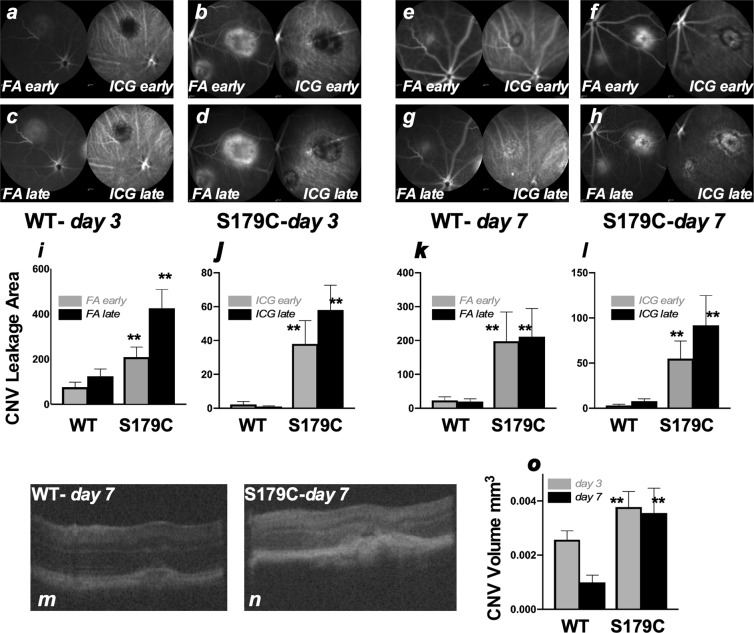
Increased laser-induced CNV leakage in TIMP3-S179C^+/+^ mutant mice. Wild-type (n = 9) and mutant *TIMP3-S179C*^+/+^ (n = 10) mice were subjected to laser burns and evaluated by SLO at day 3 and day 7. Mice were injected with NaF and ICG and imaged at “early” and “late” time points to determine leakage area as demarcated by FA- and ICGA-SLO angiography. Representative SLO images of a WT mouse imaged at day 3 (**a**) FA and ICG early (**c**) FA and ICG late (**e**) same lesions in the same WT mouse imaged at day 7 (**e**) FA and ICG early and (**g**) FA and ICG late (**b**) S179C^+/+^ mouse imaged at day 3 (**b**) FA and ICG early (**d**) FA and ICG late (**f**) same lesions in the same S179C^+/+^ mouse imaged at day 7 (**f**) FA and ICG early and (**h**) FA and ICG late. Quantitation and comparison of CNV leakage area in WT and S179C^+/+^ mice is depicted in (**i**) day 3 FA (early and late) (**j**) day 3 ICG (early and late) (**k**) day 7 FA (early and late) (**l**) day 7 ICG (early and late). Representative OCT images are presented for (**m**) WT and (**n**) S179C^+/+^ mice and CNV volume quantitated and compared (**o**). Data are presented as means ± SD of 1–3 lesions in each mouse of each genotype. Student’s two-tailed t-test **p ≤ 0.03.

We examined the induction of choroidal neovascularization (CNV) in ***Timp3***^***S1****79C****6/S1****79****C***^ mice and their WT littermates using a mouse model of laser-induced CNV. Mice were subjected to laser-induced rupture of Bruch’s membrane. Leakage of CNV lesions were imaged by scanning laser ophthalmoscopy at day 3 (Fig. 1a–d) and day 7 (Fig. 1e–h) following administration of sodium fluorescein (NaF) (FA) and Indocyanine Green (ICG) (Fig. 1a–l). Previous studies have determined that leakage of CNV in this model peaks at day 7. The leakage area of CNV lesions in ***Timp3***^***S1****79C****6/S1****79****C***^ mice (Fig. 1b,d,f,h) were significantly larger than those induced in their WT littermates (Fig. 1a,c,e,g). Quantification of CNV leakage area determined a 2-15-fold increase in lesions at day 3 and day 7 in ***Timp3***^***S1****79C****6/S1****79****C***^ mice as compared to WT controls (Fig. 1i–l). CNV leakage measured by both fluorescein angiography (FA) as well as indocyanine green angiography (ICGA) showed a significant increase in the mutant mice at both day 3 (Fig. 1j) and day 7 (Fig. 1k,l). Volume of the CNV lesions as measured by OCT (Fig. 1m,n) was also increased in mice carrying the S179C mutation (Fig. 1o).

### Increased FGFR-1 signaling in RPE-choroid tissue of *Timp3*^*S179C6/S179C*^ mice following laser-induced rupture of Bruch’s membrane

**Figure 2:**
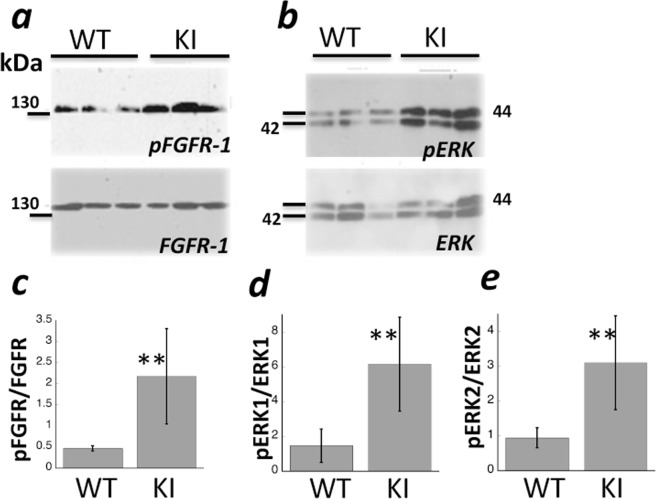
Increased FGFR phosphorylation in the RPE/choroid of S179C^+/+^ mutant mice is an early event following laser injury. RPE/choroid tissue was collected from wild-type (WT) and S179C^+/+^ mutant mice (KI) at 3 days following laser injury. Lysates were analyzed for the presence of (**a**) FGFR-1 and phosphorylated FGFR-1(pFGFR-1) and (**b**) downstream pERK. Band intensities were quantitated and compared (**c**–**e**). Data are presented as means ± SD n = 3. **p ≤ 0.05 vs WT.

To investigate whether mutant TIMP3 increased FGFR-1 signaling in a CNV mouse model, extracts of RPE-choroid tissue from WT and ***Timp3***^***S1****79C****6/S1****79****C***^ mice subjected to laser injury were prepared 3 days following laser injury and subsequently assayed for FGFR-1 expression and tyrosine phosphorylation as well as MAP kinase phosphorylation by Western blot analysis. Anti-phospho-FGFR-1 blots demonstrated that tyrosine phosphorylation of FGFR-1 was strikingly increased in ***Timp3***^***S1****79C****6/S1****79****C***^ mice compared to that in WT controls (Fig. 2a, top panel). In contrast, WT and ***Timp3***^***S1****79C****6/S1****79****C***^ mice showed similar levels of FGFR-1 protein (Fig. 2a, bottom panel). The ratios of pFGFR to FGFR protein indicated a significant increase in pFGFR following laser-injury in mutant mice. Similarly, the levels of phosphorylated ERK1/2 relative to total ERK1/2 were increased in ***Timp3***^***S1****79C****6/S1****79****C***^ mice compared with WT controls (Fig. 2b,d,e).

### Increased active MMP2 in RPE/Choroid of *Timp3*^*S179C6/S179C*^ mice following laser-induced CNV

**Figure 3:**
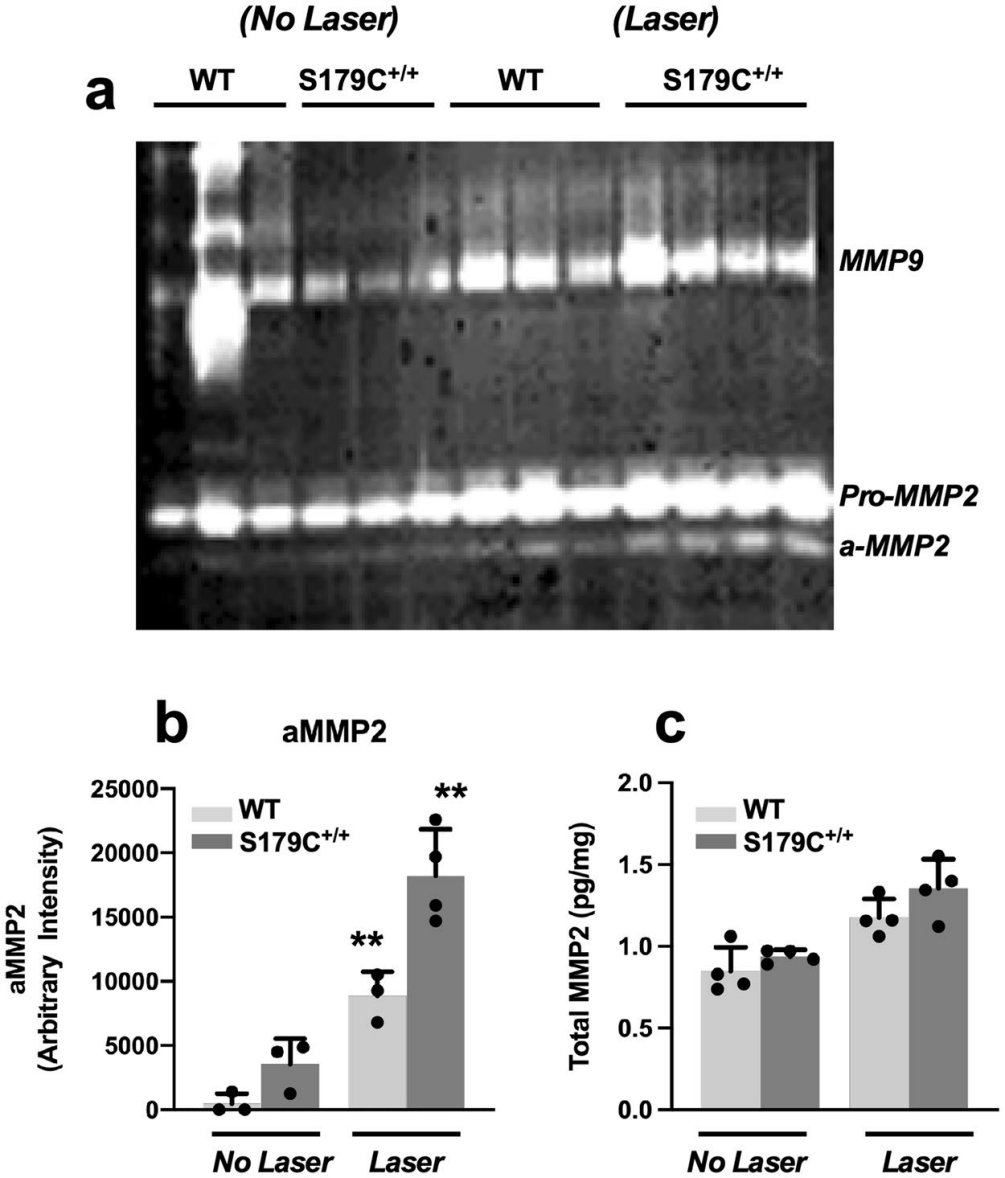
Increased MMP2 activation in RPE/choroid of S179C^+/+^ mutant mice following laser injury. RPE/choroid tissue from wild-type (WT) and S179C^+/+^ mutant mice (KI) was collected 3 days following laser injury and subjected to (**a**) gelatin zymography. Intensity of (**b**) a-MMP2 and (**c**) Total-MMP2 from RPE/choroid tissue was evaluated by ELISA and compared between WT and KI mice (n = 3–4). **p ≤ 0.04.

Since MMP2 has been implicated in CNV and the possible release of bFGF from the ECM, we examined MMP2 expression and activation in RPE-choroid tissue from WT and ***Timp3***^***S1****79C****6/S1****79****C***^ mice subjected to laser-injury. Zymography with quantitation using scanning densitometry and ELISA determined that laser-injury induced the active form of MMP2 and total MMP2 levels in WT mice, but the increase in active MMP2 was significantly higher in mutant mice when compared with non-laser-treated mice (Fig. 3). These results suggest that the presence of mutant TIMP3 in RPE may induce CNV by causing an MMP2 dependent release of bFGF by RPE resulting in increased FGFR-1 signaling in endothelial cells.

### Increased levels of bFGF and MMP2 in the conditioned medium of RPE cells expressing S179C-TIMP3

**Figure 4:**
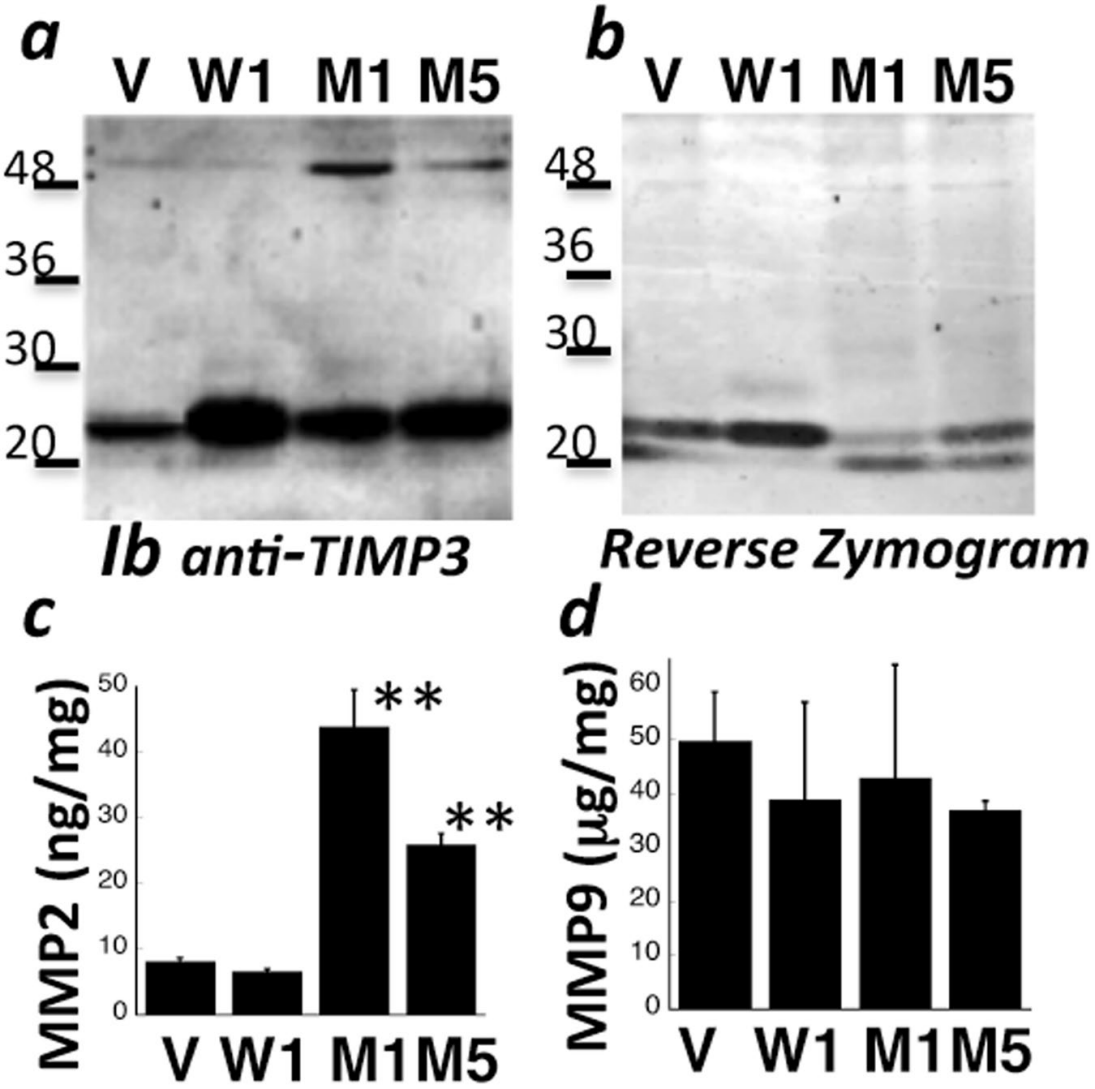
Mutant TIMP3 in RPE cells is an inefficient MMP inhibitor (**a**) Expression of TIMP3 protein was confirmed by western blot analysis in the ECM of RPE cells transfected with control vector (V), wild-type TIMP3 (W1) and S179C-TIMP3 (M1, M5). (**b**) MMP inhibitory activity of wild-type and mutant TIMP3 in the ECM of RPE cells was determined by reverse zymography (**c**) MMP2 and d) MMP9 levels were quantitated in the conditioned medium of RPE cells by ELISA. Data are expressed as means ± SD (n = 3). **p ≤ 0.01.

**Figure 5:**
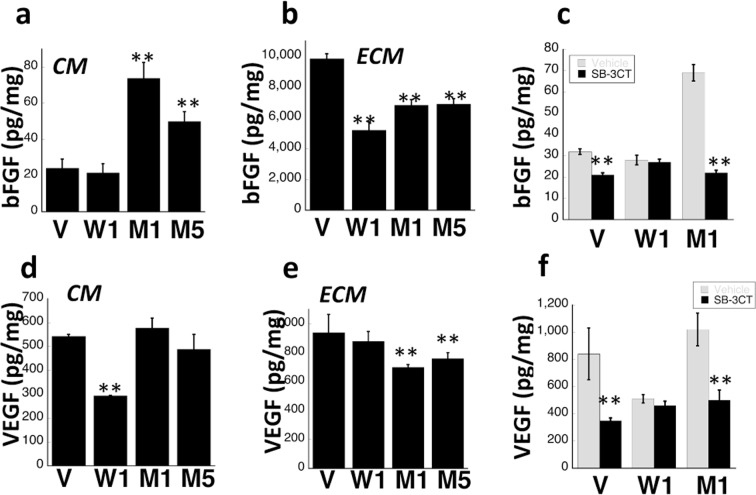
Increased bFGF in the CM of RPE cells expressing mutant TIMP3 is mediated by MMPs. ELISA was used to quantitate bFGF levels in the (**a**) CM and (**b**) ECM of RPE cells expressing control empty vector (V), wild-type TIMP3 (W1) or mutant TIMP3 (M1,M5) (**c**) Effect of an MMP2/9 inhibitor (SB-3CT, 5 μg/ml for16 hours) on bFGF secretion was evaluated in RPE cells expressing wild-type or mutant TIMP3. VEGF levels were quantitated in the (**d**) CM and (**e**) ECM of RPE cells expressing control empty vector (V), wild-type TIMP3 (W1) or mutant TIMP3 (M1,M5). Effect of MMP2/9 inhibitor (SB-3CT, 5 μg/ml for16 hours) on VEGF secretion was evaluated in RPE cells. Data are expressed as means ± SD (n = 3). **P ≤ 0.01 vs Vector.

**Figure 6:**
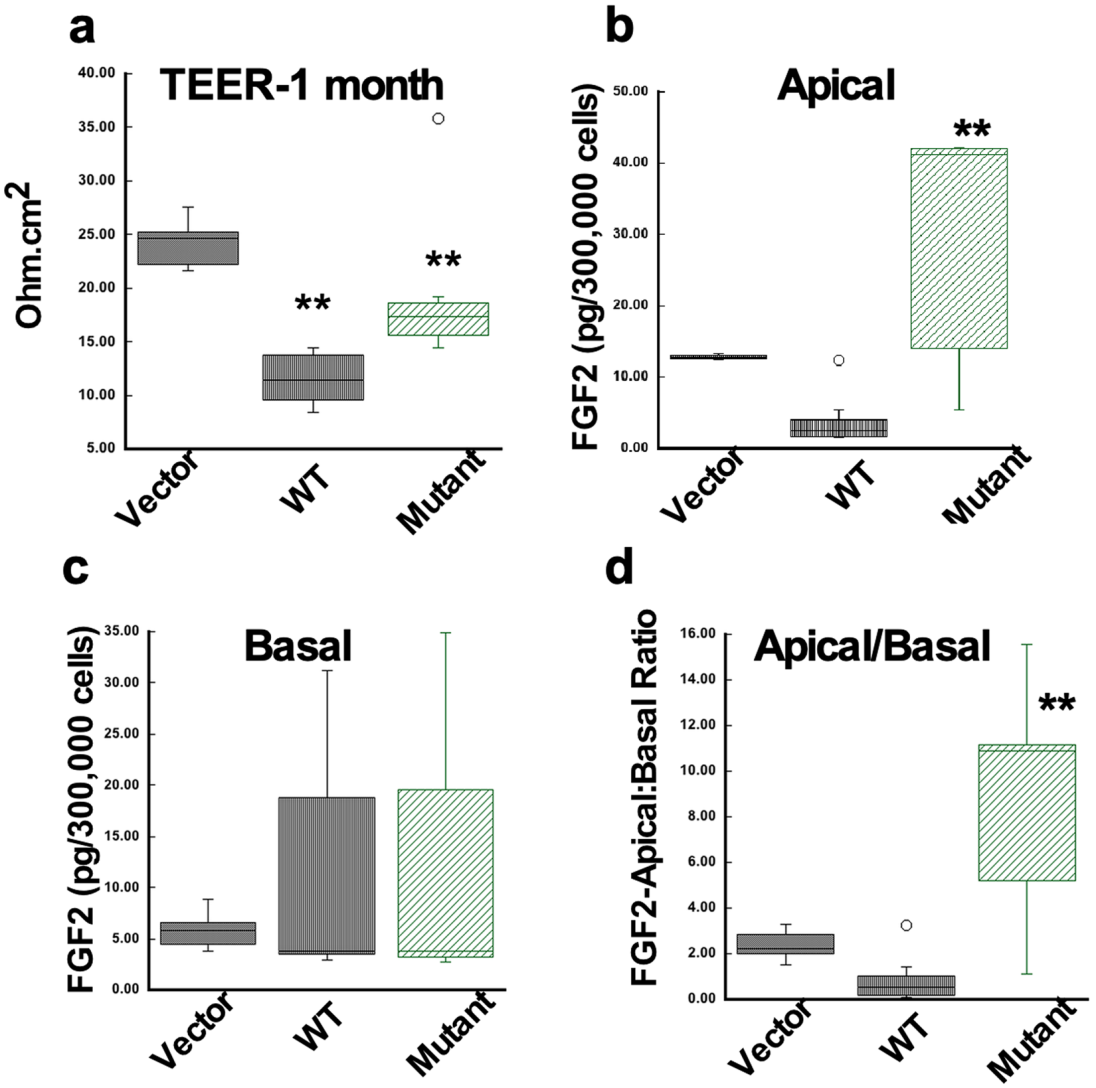
Decreased TEER and increased apical secretion of bFGF in RPE cells expressing mutant TIMP3. Box and whisker plots depict comparisons between RPE cells expressing empty vector (Vector), wild-type TIMP3 (WT) or mutant S179C-TIMP3 (Mutant) of (**a**) TEER (**b**) FGF2 concentration in the apical conditioned medium (**c**) FGF2 concentration in the basal conditioned medium (**d**) ratio of apical/basal FGF2 (n = 6–8). **p ≤ 0.01 vs Vector.

It has been suggested that expression of S179C-TIMP3 at a relatively low-level mimics the heterozygous autosomal dominant phenotype^49^. To determine the mechanism(s) underlying mutant TIMP3-induced angiogenesis in SFD, we generated stable RPE cell lines expressing equivalent amounts of WT (W1) or S179C-TIMP3 (M1 and M5). Western blot analysis determined a ~2.5 fold increase of WT-TIMP3 levels in the ECM of W1 and 1.8–2.2 fold increase of mutant-TIMP3 levels in M1 and M5 RPE cell lines (Fig. 4a) when compared with cells transfected with empty vector (V). MMP inhibitory activity of mutant-TIMP3 in the ECM of M1 and M5 cell lines was reduced compared with WT-TIMP3 (W1), as determined by reverse zymography (Fig. 4b). To evaluate if this loss of MMP inhibitory activity was associated with increased amounts of secreted MMPs, the conditioned medium (CM) from these RPE cells was analyzed for MMP2 and MMP9 using ELISA. Total MMP2 levels (pro- and active MMP2), were increased two to four-fold in cells expressing mutant TIMP3 relative to vector controls (Fig. 4c). Interestingly, there was no difference in secreted MMP9 levels in mutant cells (Fig. 4d). These results confirm our previous findings with independent cell lines^49^. Since it has been hypothesized that bFGF is secreted by RPE cells in association with CNV^52,53^}, we initially measured the levels of bFGF in the CM and the ECM of RPE cells grown on plastic tissue culture dishes using ELISA. The levels of bFGF were significantly increased in the CM (Fig. 5a) and decreased in the ECM (Fig. 5b) of RPE cells expressing mutant TIMP3 (M1, M5) when compared with control vector cells (V). Cells over-expressing wild-type (W1) TIMP3 on the other hand did not show a significant increase in the conditioned medium relative to control vector cells. These results suggest that the presence of mutant TIMP3 may result in a release of ECM bound-bFGF into the CM. While WT TIMP3 results in a decrease of bFGF in the ECM it is not associated with an increased release into the CM. In contrast, the levels of VEGF in the CM of mutant (M1 and M5) cells remain unchanged in spite of a significant decrease in the ECM, suggesting that VEGF levels in the CM may not be solely derived from release of ECM-bound VEGF and other factors might be in play. Since MMPs may regulate angiogenic factor release from the ECM^54–56^, we investigated the effect of a selective MMP2/9 inhibitor (SB-3CT) on the release of bFGF and VEGF in RPE cells. We found that SB-3CT decreased bFGF (Fig. 2c) and VEGF (Fig. 2f) levels in the CM of mutant cells (M1 and M5) but not in cells expressing wild-type TIMP3 (W1). These results suggest that MMP2 may play a role in the release of bFGF and/or VEGF from ECM in mutant RPE cells. Recent studies suggest that long term polarized culture of RPE cells on transwells more closely mimics RPE cells *in vivo*. To evaluate if there was a change in the polarized secretion of bFGF in RPE cells expressing mutant TIMP3 we quantitated the amount of bFGF secreted into the apical and basal CM of cells plated on transwell filters and cultured for 1 month. While there was a loss of trans-epithelial electrical resistance (TEER) in cells overexpressing WT and mutant TIMP3 (Fig. 6a), cells expressing mutant TIMP3 showed a significant increase in apical FGF secretion compared with cells expressing WT TIMP3 (Fig. 6b,c). There was no apparent change in ZO-1 staining of mutant RPE cells (data not shown). The ratio of apical to basal FGF secretion was also significantly increased in RPE cells expressing mutant TIMP3 (Fig. 6d).

### RPE cells expressing S179C-TIMP3 induce increased bFGF-dependent tube formation in endothelial cells

**Figure 7:**
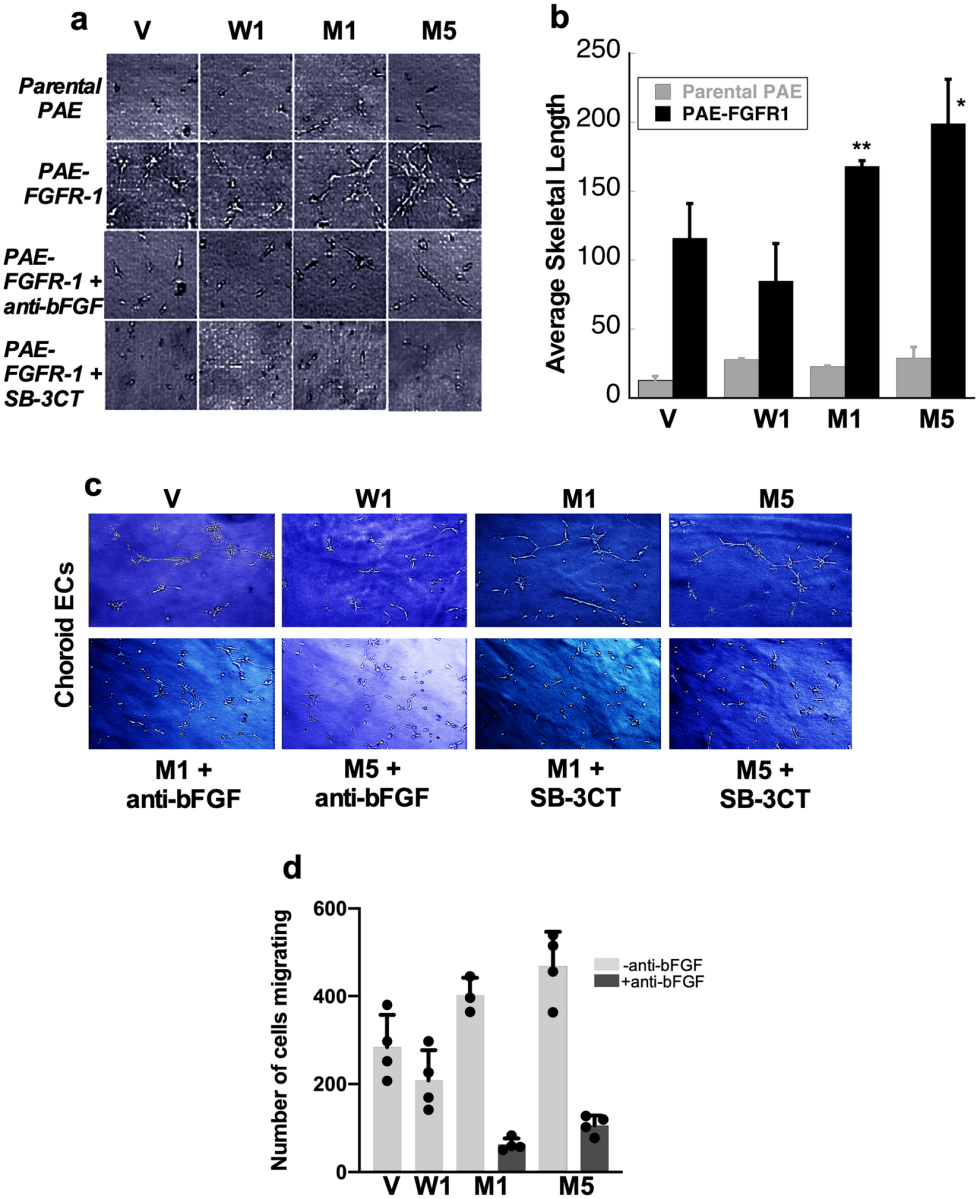
Increased induction of endothelial cell tube formation by CM of RPE cells expressing S179C-TIMP3 is mediated by bFGF. (**a**) Parental porcine aortic endothelial cells (Parental PAE, top panel) and PAE expressing FGF receptor-1 (PAE/FGFR-1, 2^nd^ panel) were embedded in three-dimension (3D) collagen gels and incubated with conditioned medium (CM) of RPE cells expressing empty vector (V), wild-type TIMP3 (W1) or S179C-TIMP3 (M1, M5) in the presence of 10 μg/ml anti-bFGF neutralizing antibody (third panel) or a MMP2/9 inhibitor (SB-3CT, 5 μg/ml) for 48 hours. Differentiated cells were photographed using an inverted microscope (original magnification 200x) and (**b**) quantitated using customized macros generated in Image-Pro Plus 5.1. Data are presented as means ± SD n = 3. *p ≤ 0.05 vs vector control **p ≤ 0.01 vs vector control. (**c**) RF6A monkey choroid endothelial cells were subjected to tube formation assay in the presence of conditioned medium (CM) of RPE cells expressing empty vector (V), wild-type TIMP3 (W1) or S179C-TIMP3 (M1, M5) in the presence of anti-bFGF or SB-3CT (lower panel) and compared to the absence of inhibitors (upper panel). (**d**) Migration of RF6A choroidal endothelial cells towards conditioned medium of RPE cells expressing empty vector (V), wild-type TIMP3 (W1) or S179C-TIMP3 (M1, M5). Migrating cell number is expressed as mean +/− SD of quadruplicate samples.

We investigated whether RPE cells expressing mutant TIMP3 could promote angiogenesis via bFGF, To this end we examined the ability of the CM of RPE cell transfectants to induce EC tube formation, in porcine aortic endothelial (PAE) cell lines that were engineered to express FGFR-1 (PAE-FGFR-1) and compared the response with that induced in cells that lacked FGFR-1 (Parental PAE). PAE cells were grown in 3-D collagen gels, and incubated with serum free CM from RPE cell lines over two days. As shown in Fig. 7, the CM from the vector transfected control RPE cells (V) induced tube-like structures in PAE/FGFR-1 cells, but not in parental PAE cells which indicated that the angiogenic tube formation response was mediated via FGF/FGFR1 signaling. CM from RPE cells transfected with WT-TIMP3 (W1) showed reduced tube formation while CM from mutant-TIMP3 cells (M1, M5) induced an extensive tubular network with significantly increased number and length of EC tubes. Quantitation of tube formation (Fig. 7b) identified a 40–50% increase in the average skeleton length of tubes formed by PAE/FGFR-1 cells treated with the mutant cell-CM, but a 20% decrease in tube length by CM from WT cell-CM relative to the vector-CM (Fig. 3). Inclusion of neutralizing antibody against bFGF (5 μg/ml) in the CM blocked tubular responses induced by the CM from all RPE cell transfectants (Fig. 7, PAE-FGFR-1 + anti-bFGF). In addition, treatment of PAE/FGFR-1 cells with a selective MMP2/9 inhibitor (SB-3CT) also inhibited tube formation induced by the CM from all RPE cell transfectants, indicating this process to be MMP dependent. This, together with the absence of induction of tube formation in parental PAE cells lacking FGFR, confirms that the *in vitro* angiogenic response is mediated via a bFGF-FGFR-1 pathway that is MMP2 dependent. Interestingly, the CM of cells expressing mutant TIMP3 also induced increased tube formation but to a lesser extent in PAE cells expressing VEGFR2 (PAE/KDR) cells, suggesting that VEGF may also play a role in this process (data not shown). Choroid endothelial cells exposed to CM showed similar tube formation responses (Fig. 7c) as the PAE-FGFR-1 cells. Conditioned medium from RPE cells expressing mutant TIMP3 (M1,M5) induced increased migration of choroid endothelial cells when compared to CM from RPE cells expressing wild-type TIMP3(W1) or empty vector (V) that appeared to be inhibited by anti-bFGF antibody (Fig. 7d).

## Discussion

SFD is an autosomal dominant, fully penetrant degenerative disorder of the macula that is caused by specific mutations in the TIMP3 gene^57–65^. The disease usually manifests with symptoms of night blindness or sudden loss in visual acuity in the third to fourth decades of life due to recurrent CNV^43,66^. We have previously reported TIMP3 to be a potent angiogenesis inhibitor^29,36,67^ and determined that S179C-TIMP3-related angiogenesis *in vitro* and *in vivo* might be a result of reduced MMP inhibitory activity with a concomitant increase in MMP2 activity^48^ in endothelial cells. We also identified the ability of S179C-TIMP3 to increase the amount of VEGFR-2 on the surface of endothelial cells by an unidentified post-transcriptional mechanism to induce an angiogenic phenotype^48^. In this study, we demonstrate that S179C-TIMP3 may also induce angiogenesis via a FGFR-1 dependent pathway by increasing the release of ECM-bound bFGF and subsequent bFGF activity. This is based on the findings that 1) expression of SFD-related S179C-TIMP3 mutation in human RPE cells leads to increased levels of bFGF in the CM with a decrease in the ECM, 2) exposing ECs expressing FGFR-1 to the CM from mutant RPE cell resulted in increased migration and tube formation with a bFGF dependency 3) increased bFGFR signaling in the RPE/Choroid of mutant mice subjected to laser injury to induce CNV.

A number of early studies have demonstrated that the FGFR pathway cross talks with other receptor tyrosine kinases such as VEGFR2 in a variety of physiological and pathological processes. Murakami *et. al*.^68^ elegantly demonstrated that FGF driven regulation of VEGFR2 expression is required for angiogenesis and adult arteriogenesis. Our previous results showing increased VEGFR2 expression on endothelial cells expressing mutant TIMP3 together with results from the current study, which identifies increased release of bFGF from RPE cells expressing mutant TIMP3 suggests that this crosstalk maybe critical for the pathogenesis of CNV in SFD.

One question that is raised by these findings is how S179C-TIMP3 increases bFGF mobilization from the ECM. It has been previously shown that bFGF binds to a cell surface- and ECM-associated heparan sulfate proteoglycan (HSPG)^18^. The HS-immobilized FGF can be released by heparanases^69^ termed as endoglycosidases that cleave the heparan sulfate glycosaminoglycan from proteoglycan core proteins and degrade them to small oligosaccharides^70^. Unpublished data from our laboratory suggests that the expression of WT- TIMP3 in RPE cells leads to an increase in HS content in the ECM with a concomitant decrease in the CM. On the other hand, expression of mutant S179C-TIMP3 in RPE cells decreases HS content in the ECM with a corresponding increase in HS levels in the CM. This indicates that WT TIMP3 can inhibit HS degradation in the ECM, allowing bFGF to remain sequestered in the ECM. On the other hand, mutant TIMP3 promotes HS degradation in the ECM, leading to increased bFGF release. Studies in the literature suggest that shedding of syndecan ectodomains containing HS chains is mediated by a TIMP3-sensitive metalloproteinase^32^. It could be hypothesized that S179C-TIMP3 may upregulate this TIMP3-sensitive metalloproteinase activity, leading to increased HS. Another possibility is that mutant TIMP3 increases HS degradation by regulating heparanase activity. These possibilities warrant testing in the future. In addition to HSPG, MMPs have been proposed to regulate angiogenesis by releasing ECM-bound angiogenic factors such as bFGF through the degradation of a variety of ECM components^25,26^. Furthermore, a selective MMP2 inhibitor reduced bFGF release from RPE cells expressing mutant TIMP3. We noted that increased bFGF release was associated with MMP2 secretion when mutant TIMP3 was expressed in RPE cells at a relatively low level but not at relatively high levels (data not shown), which implies that the MMP inhibitory activity may be reduced and not completely absent in mutant RPE cells. On the other hand, it might also suggest that MMP2 is involved but not essential for bFGF release from the ECM. In an RNA sequencing analysis of RPE and choroid from wild-type and mutant mice we found no change in the expression of FGF (data not shown) which suggests that the increase in FGF in the conditioned medium is likely to be due to release from cells. In polarized RPE cells we also did observe an increase in apical FGF in cells expressing mutant TIMP3. Given that there was decreased TEER in cells expressing both wild type and mutant TIMP3 it is unlikely that any change in apical/basal ratios of growth factors is due to loss of barrier function. However, future detailed studies on the outer BRB will help confirm this.

Of the TIMP family members, TIMP3 has a very broad inhibitory profile that includes in addition to a wide range of MMPs, members of the related ADAMs (A Disintegrin and Metalloprotease) and ADAMTSs (ADAM with Thrombospondin motifs). Unpublished results in the laboratory determined a reduction in ADAM17 protein in the choroid/RPE of S179C mice by western blot analysis. While we did not perform a comprehensive investigation of expression of all the known TIMP3 substrates in the choroid tissue of mutant mice this would be an important analysis to conduct in the future. In addition, while we do see an increase in active MMP2 in the choroid/RPE of mutant mice, there appears to be no change in the pro-MMP2 levels. This suggests, that the presence of S179C TIMP3 in the choroid/RPE results in either an increase in activation of pro-MMP2 to active MMP2 or a loss of inhibition of activation of MMP2.

At this point we cannot say for certain that there is a direct association between the loss of TIMP3 function, increase of active MMP2 and increased bFGF signaling as the data suggests a correlation. The results in Fig. 5 suggests that the increase in the secretion of bFGF from RPE cells expressing the S179C mutation (M1) can be inhibited with an MMP inhibitor (SB-3CT) indicating likely mediation by MMPs. Since overexpression of wild-type TIMP3 does not induce an increase in the secretion of bFGF one can draw a reasonable conclusion that expression of mutant TIMP3 in RPE cells likely leads to an increase in active MMP2. However, it must be noted that while SB-3CT inhibits MMP2 with a Ki of 13.9 nM it also has the capability of inhibiting MMP9, albeit with a higher Ki (600 nM). In the absence of specific MMP2 inhibition there is however, there is still a possibility that other mediators/proteases may play a role. Another confounding observation is that MMP9 is present at significantly higher levels in RPE cells compared with MMP2. It will be important to ascertain how small changes in MMP2 can regulate changes in bFGF secretion in the presence of large amounts of MMP9 which are likely to have similar effects of growth factor release from the matrix.

One question that arises from these studies is whether S179C-TIMP3 can enhance bFGF activity. It has been suggested that the interaction of FGFs with HSPGs increases their receptor binding affinity by stabilizing growth factor-receptor complexes, and HSPGs and heparin may facilitate FGFR dimerization and subsequent activation^15,21,22^. In addition, physiological degradation by heparanase has been reported to convert the soluble syndecan-1 ectodomain from an inhibitor to a potent activator of FGF-2^69^.

There has been much debate on whether SFD pathogenesis is due to a loss-of-function or a gain-of-function mutation in TIMP3^48,50,61,71,72^. While dimerization of TIMP3 has been reported by others and hypothesized to be a consequence of an unpaired cysteine and aberrant disulfide bond formation, we did not observe consistent dimerization of the mutant protein in the choroid RPE. This could be a consequence of age, sex or other confounding factors that we have not been analyzed systematically but could potentially provide some useful insights. Our previous studies have demonstrated that expression of S179C-TIMP3 in ECs induced increased angiogenesis by the up- regulation of VEGFR-2- protein levels, suggesting a gain of function^48^. In the present study, we demonstrate that mutant TIMP3 may induce increased angiogenesis via a FGFR-1-dependent pathway by increased bFGF release and activity rather than regulation of FGFR-1 receptor protein levels. Since the exact mechanism of increased release of bFGF by mutant TIMP3 and the role of WT-TIMP3 in the regulation of this process is unknown, we are unable to completely rule out a loss of function phenotype. Our studies on the *TIMP3-S179C*^+/+^ mutant mice clearly demonstrate that these mice are more susceptible to laser-induced CNV. Under physiological conditions however, *TIMP3-S179C*^+/+^ knock-in mice display only slight abnormalities in the inner aspect of Bruch’s membrane and RPE basic microvilli^51^. In our previous and present studies, the presence of mutant protein in RPE/choroid of *TIMP3-S179C*^+/+^ knock-in mice showed mild changes in the expression of proangiogenic proteins such as MMP2, bFGF and VEGFR-2 in spite of altered molecular and functional features of mutant TIMP3. An absence of typical SFD-related pathology in the mutant mice such as thickening of Bruch’s membrane, atrophy of the choriocapillaris and spontaneous CNV, and significant increases of pro-angiogenic proteins seen in cultured cells like upregulation of VEGFR-2 and bFGF levels lead one to speculate that either the short life span of the mouse may not allow sufficient accumulation of the protein to generate pathology or transgenic mice adapt to molecular or functional changes via a compensatory mechanism. Alternatively, it is possible that the TIMP3 mutation makes the retina or choroid/RPE more susceptible to an environmental or epigenetic stress, which may be absent in the inbred mouse strain housed under controlled conditions of the vivarium. Following laser-induced injury, *TIMP3-S179C*^+/+^ knock-in mice exhibit increased CNV with a striking increase in the auto-phosphorylation of FGR-1, and increased MMP2 activity during the early phase of CNV when compared with their WT littermates. Similar findings of increased CNV following laser injury were also observed in TIMP3 knockout mice, which suggests a possible loss of function^44^.

While inhibiting VEGF has significantly changed the therapeutic landscape of age-related macular degeneration (AMD), a significant number of patients do not respond to these treatments. This suggests that other factors play a role in the pathophysiology of choroidal neovascularization (CNV), which is the leading cause of vision loss in AMD. Early this year, the AMD consortium identified rare coding variants in the TIMP3 gene when analyzing 16,144 patients and 17,832 controls^73^. In addition, they identified the first genetic association signal specific to wet AMD near MMP9. The clinical and histopathological similarities between AMD and SFD and the identification of variants in the TIMP3 and matrix metalloproteinase pathway in AMD suggest that similar downstream effectors might be in play in both conditions. We have used the model of Sorsby Fundus Dystrophy, a monogenic disease in which the majority of patients develop CNV, to understand the molecular mechanisms that cause abnormal neovascularization. We conclude that bFGF may be an important regulator of ocular angiogenesis in SFD and could be a potential therapeutic target in SFD and possibly AMD.

## Methods

### Mice

Heterozygous *Timp3*^*+/S179C*^ were crossed to generate homozygous *Timp3*^*S179C6/S179C*^ mice and their age-matched littermate controls. All experimental procedures were conducted according to the ARVO statement for the Use of Animals in Ophthalmic and Vision Research.

### Induction and quantification of CNV

All animal studies were approved by the Animal Care and Use Committee (IACUC) guidelines of the Cleveland Clinic and conformed to the National Institutes of Health Guide for the Care and Use of Animals in Research and the ARVO statement for the use of animals in ophthalmic and vision research. CNV was induced in mice following laser injury of Bruch’s membrane by four burns using green argon laser as described in previous studies^44,74^. Briefly, mice were anesthetized intra-peritoneally with 65–68 mg/Kg sodium pentobarbital. Topical anesthesia was achieved by instilling 0.5% proparacaine solution to the cornea. Following anesthesia, pupils were dilated with 0.5% topical tropicamide/phenylephrine combination drops (Santen Pharmaceuticals, Osaka, Japan).

Four laser spots were placed in the superior, superior-temporal, or superior-nasal quadrants of the fundus using a green solid-state laser (532 nm; 150–200 mW; 0.100 second pulse duration; 75 μm spot size) using a slit lamp delivery system and a microscope coverslip placed affixed to the cornea using a drop of Systane Ultra artificial tears (Alcon, Ft Worth, TX). Lesions were imaged at day 3 and day 7 by scanning laser ophthalmoscopy (SLO) using a Model HRA2 (Heidelberg Engineering, Carlsbad, CA). In most cases baseline images were collected prior to laser injury to ensure normal retinas. Images were collected in “R’ reflectance mode via the Red Free-Dark field (RFDF) and Infrared-dark field (IRDF) and used to quantitate lesion size at day 3. Following injection of sodium fluorescein (NaF) and indocyanine green (ICG), images were collected using the “A” angiography mode at early and late time-points following injection of dye. The early phase captures filling of the retinal arteries, arterioles and capillaries while the late phase includes the filling of the veins. ICG is a dye that fluoresces in the infra-red (non-visible) light the wavelength of which has the ability to penetrate the retinal layers making the choroid vasculature visible under infra-red sensitive conditions. The HRA-2 system was operated under Heidelberg Eye Explorer software v1.7. Equipped with a 55° wide-field objective lens, the system provides a posterior pole field of view of approximately 1.5 mm in mice^75^. The system was operated in high-resolution mode, which provides an image pixel format of 1536 × 1536 with the wide-field lens. CNV images were analyzed in ImageJ by measuring area of leakage. This value was then normalized for size of laser burn measured at day 3 in IRDF images. Ultra high-resolution spectral domain optical coherence tomography (OCT) system was used for *in-vivo* cross-sectional imaging (Bioptigen, Leica Microsystems, Germany). Lesion volume was measured from B scans of OCT images. By assuming CNV lesion as an ellipsoid, lesion volume were obtained by measuring length, depth, and width of each lesion in OCT rectangular volumetric scans as described^76^. The B-scan was used to measure the width (a) and depth (b) of the lesion. The volume intensity projection image was used to measure the length (c) of the lesion. The lesion volume was calculated using the formula ${\rm{A}}=\frac{4}{3}\pi {\rm{abc}}$.

### Cells and reagents

Human RPE cells (ARPE-19 cell line) expressing wild-type (WT) or S179C mutant TIMP3 (M) or vector alone (V) were grown on polyester filters (Transwell filters; Costar; pore size 0.4μm) or plastic tissue culture dishes in 1% FBS for 1–3 months. Media changes were performed twice a week and prior to experiments cells were washed in serum free medium and serum starved for 48 hours. Porcine aortic endothelial cell lines expressing FGFR-1 (PAE/FGFR-1)^77^ were a kind gift from Lena Claesson-Welsh (Department of Genetics and Pathology, Rudbeck Laboratory, Uppsala, Sweden). Cells were cultured in Ham’s F-12/DMEM medium supplemented with 10% fetal calf serum (FCS) (Cambrex, East Rutherford,NJ), 50 unit/ml penicillin and 50 μg/ml streptomycin as described^36^.ARPE-19 cell lines were authenticated using short -tandem repeat (STR) analysis and were found to be an exact match to ATCC cell line CRL-2302 (ATCC). Recombinant human FGF basic (157 aa) was purchased from R & D Systems (Minneapolis, MN). Antibodies: Anti-bFGF antibody (neutralizing) (EMD Millipore/Calbiochem, Darmstadt Germany); Monoclonal anti-TIMP3 antibody (EMD Millipore/Chemicon, Temecula CA); FGFR1 rabbit polyclonal antibody and phosphor FGFR1 rabbit monoclonal antibody (Epitomics, Inc. Burlingame, CA); Anti-MAP kinase and anti-MAP kinase, phospho-specific rabbit polyclonal antibodies (EMD Millipore/Calbiochem, Darmstadt Germany). MMP2/MMP9 inhibitor IV (SB-3CT) was from Chemicon, Temecula, CA. Matrigel and type 1 collagen were purchased from Collaborative Biomedical Products, Bedford, MA.

### Generation of human RPE cells expressing wild-type or S179C mutant TIMP3

A 550 bp TIMP3 insert from a human cDNA clone was cloned into expression vector pCEP4 (Invitrogen)^48^. ARPE-19 cells were transfected with wild-type TIMP3 or S179C-TIMP3 cDNA in pCEP4 using Effectene® Transfection Reagent (Qiagen, Hilden Germany) according to the manufacturer’s protocol. Stable clones were isolated by Hygromycin selection. Control cells were transfected using pCEP4 vector without any insert and selected with Hygromycin. Western blot analysis and reverse zymography confirmed expression of TIMP3.

### Preparation of ECM and conditioned medium

Media was removed and replaced with serum-free medium, and incubated for 2 days. Following collection of the conditioned medium (CM), cells were removed following a 10 min incubation in Ca2+-, Mg2+-free phosphate buffered saline (PBS) containing 2.5 mM EDTA. After several rinses in PBS and water, the ECM was scraped in a small volume of electrophoresis sample buffer without reducing agent.

### Zymography and reverse zymography

Equal amounts of non-reduced samples were loaded onto a 7.5% Bis-Arylamide gel with 1 mg/ml gelatin (zymography) or onto a 12% gel plus conditioned media from RPE cells treated with 100 nM PMA, as a source of MMPs, for reverse zymography. Following electrophoresis, gels were processed as described previously^49^. Briefly, gels were agitated in a solution of 25 mg/ml Triton X-100 to remove SDS and to promote re-naturation of proteases and inhibitors. The Triton X-100 was washed off with water and the gels then incubated for 16 hours in 50 mM Tris-HCl (pH 7.5) containing 5 mM CaCl_2_ and 0.2 mg/ml sodium azide at 37 °C. Gels were stained with 5 mg/ml Coomassie Blue R-250 in acetic acid/methanol/water (1:3:6) for 1–2 hours and de-stained with acetic acid/methanol/water (1:3:6).

### Western blotting

Cells were collected and lysed in buffer composed of 20 mM Tris-HCl, pH 7.5, 150 mM NaCl, 2.5 mM EDTA, 10% glycerol, 1% Triton X-100, and protease inhibitor cocktail tablets (Roche Life Sciences, Indianapolis, IN). The posterior cups of mouse eyes (RPE-choroid complex without the neural retina) were dissected and homogenized in the same lysis buffer. Protein concentration was determined using a micro-BCA protein assay according to the manufacturer’s (Bio-Rad, Hercules, CA) instructions. Equal protein amounts were separated by SDS-PAGE, transferred to nitrocellulose membranes and subsequently probed with the indicated antibodies. After washing with PBS/Tween, proteins were detected with HRP-conjugated second lgG antibodies followed by ECL. The blots were stripped with Western Re- Probe^TM^ solution (G-Biosciences, St Louis, MO) for 30 minutes and reprobed. Band densities were compared using ImageJ (http://rsb.info.nih.gov/ij/index.html) using the gel analysis mode after converting the image to a gray-scale image. Density of bands is always expressed relative to control actin bands from the same membrane following stripping and reprobing.

### Enzyme-linked immunoassay (ELISA)

FGF-2 was measured by multiplex array (Eve Technologies, Calgary CA). Total FGF secreted into the apical and basal media by 100,000 cells plated was calculated based on media volumes in the apical and basal chambers of the transwells respectively. Data is represented as box plots (n = 5–8).

### Endothelial cell migration

A modified Boyden chamber assay was used to evaluate the migration of RF6A choroidal endothelial cells as described previously^36,48^. 8.0μm pore PVDF polycarbonate membranes were precoated with collagen type 1 (100 μg/ml). Conditioned medium from RPE cells expressing empty vector (V), wild-type TIMP3 (W1) or S179C-TIMP3 (M1, M5) was placed in the lower wells. RF6A cells (3 × 10^5^) were placed in the upper wells and allowed to migrate towards the conditioned medium in the lower wells. Chambers were incubated for 4 hours at 37 °C in a 5% CO2 humidified incubator. Cells on top of the filter were removed with a scraper. Bottom surface of the filter containing migrated cells was fixed and stained with hematoxylin prior to being mounted on a glass slide. Number of migrated cells was counted in each well and standard deviation calculated from quadruplicate samples.

### Tube formation assay

*In vitro* formation of tubular structure was examined within a modified three-dimension (3-D) collagen matrix as previously described. Briefly, Type 1 collagen was mixed with 10X DMEM and 0.1 M NaOH (8:1:1), adjusted to a solution containing 10 μg/ml growth factor reduced matrigel matrix (BD, Biosciences), and then distributed in 48-well dishes. After gelling, serum-starved cells were seeded on the collagen gels at 1.5 × 10^4^ cells/well, and stimulated or not with bFGF (50 ng/ml) or the conditioned medium from RPE cell transfectants in the presence or absence of anti-bFGF (neutralizing) antibody (10–30 μg/ml) or a selective MMP2 inhibitor (1–5 μg/ml). After 4 h of incubation at 37 ^o^C, a second layer of collagen was added, and cultures were kept under the same conditions as described above. Tube formation was examined using phase-contrast microscopy at the indicated time points, and quantitated with customized macros generated in Image-Pro 5.0.

### Statistical analysis

The *p* values were calculated using Students t test by comparisons with control samples tested at the same time.
